# Radiomic and Artificial Intelligence Analysis with Textural Metrics Extracted by Contrast-Enhanced Mammography and Dynamic Contrast Magnetic Resonance Imaging to Detect Breast Malignant Lesions

**DOI:** 10.3390/curroncol29030159

**Published:** 2022-03-13

**Authors:** Roberta Fusco, Elio Di Bernardo, Adele Piccirillo, Maria Rosaria Rubulotta, Teresa Petrosino, Maria Luisa Barretta, Mauro Mattace Raso, Paolo Vallone, Concetta Raiano, Raimondo Di Giacomo, Claudio Siani, Franca Avino, Giosuè Scognamiglio, Maurizio Di Bonito, Vincenza Granata, Antonella Petrillo

**Affiliations:** 1 Medical Oncolody Division, Igea SpA, 80013 Naples, Italy; r.fusco@igeamedical.com (R.F.); e.dibernardo@igeamedical.com (E.D.B.); 2 Department of Electrical Engineering and Information Technologies, Università degli Studi di Napoli Federico II, 80125 Naples, Italy; adelepiccirillo@gmail.com; 3 Radiology Division, Istituto Nazionale Tumori-IRCCS-Fondazione G. Pascale, 80131 Naples, Italy; m.rubulotta@istitutotumori.na.it (M.R.R.); t.petrosino@istitutotumori.na.it (T.P.); m.barretta@istitutotumori.na.it (M.L.B.); m.mattaceraso@istitutotumori.na.it (M.M.R.); p.vallone@istitutotumori.na.it (P.V.); c.raiano@istitutotumori.na.it (C.R.); a.petrillo@istitutotumori.na.it (A.P.); 4 Senology Surgical Division, Istituto Nazionale Tumori-IRCCS-Fondazione G. Pascale, 80131 Naples, Italy; r.digiacomo@istitutotumori.na.it (R.D.G.); c.siani@istitutotumori.na.it (C.S.); f.avino@istitutotumori.na.it (F.A.); 5 Pathology Division, Istituto Nazionale Tumori-IRCCS-Fondazione G. Pascale, 80131 Naples, Italy; g.scognamiglio@istitutotumori.na.it (G.S.); m.dibonito@istitutotumori.na.it (M.D.B.)

**Keywords:** contrast-enhanced mammography, magnetic resonance imaging, image enhancement, contrast media, radiomics, artificial intelligence

## Abstract

**Purpose**:The purpose of this study was to discriminate between benign and malignant breast lesions through several classifiers using, as predictors, radiomic metrics extracted from CEM and DCE-MRI images. In order to optimize the analysis, balancing and feature selection procedures were performed. **Methods**: Fifty-four patients with 79 histo-pathologically proven breast lesions (48 malignant lesions and 31 benign lesions) underwent both CEM and DCE-MRI. The lesions were retrospectively analyzed with radiomic and artificial intelligence approaches. Forty-eight textural metrics were extracted, and univariate and multivariate analyses were performed: non-parametric statistical test, receiver operating characteristic (ROC) and machine learning classifiers. **Results**: Considering the single metrics extracted from CEM, the best predictors were KURTOSIS (area under ROC curve (AUC) = 0.71) and SKEWNESS (AUC = 0.71) calculated on late MLO view. Considering the features calculated from DCE-MRI, the best predictors were RANGE (AUC = 0.72), ENERGY (AUC = 0.72), ENTROPY (AUC = 0.70) and GLN (gray-level nonuniformity) of the gray-level run-length matrix (AUC = 0.72). Considering the analysis with classifiers and an unbalanced dataset, no significant results were obtained. After the balancing and feature selection procedures, higher values of accuracy, specificity and AUC were reached. The best performance was obtained considering 18 robust features among all metrics derived from CEM and DCE-MRI, using a linear discriminant analysis (accuracy of 0.84 and AUC = 0.88). **Conclusions:** Classifiers, adjusted with adaptive synthetic sampling and feature selection, allowed for increased diagnostic performance of CEM and DCE-MRI in the differentiation between benign and malignant lesions.

## 1. Introduction

In the screening, detection and follow-up of breast cancer, the mammography (MX) was considered the first imaging examination [1,2]. In particular, thanks to the technological improvements achieved by combining digital mammography with techniques that allow low and high energy images to be obtained, and with the administration of iodate contrast agent, it is possible to acquire images that emphasize the vascularity linked to malignant lesions by the contrast agent enhancement. This imaging technique is recognized as contrast-enhanced mammography and exploits the same physiological mechanisms as dynamic contrast-enhanced magnetic resonance imaging (DCE-MRI).

DCE-MRI is an important complementary diagnostic imaging technique that was validated in the screening of high-risk women and dense breasts and in the monitoring of oncological therapies, thanks to its capability of combining morphological and functional information [2,3].

Previous studies have evaluated the sensitivity of CEM compared to conventional digital MX, ultrasound (US) and MRI [4,5,6,7,8]. CEM sensitivity has been reported in the range of 90–100% [5,6,7], which is significantly higher than the sensitivity of MX or US alone [4,5,6,7]. Moreover, CEM allows for the identification of additional occult cancers via mammography to more accurately assess the disease extent, and to guide surgical and treatment planning [8,9,10,11,12].

Radiomics and artificial intelligence approaches have been extensively applied to process both CEM and DCE-MRI in order to increase diagnostic performance in the detection of malignant breast lesions [13,14]. By means of the radiomics approach, it is possible to obtain, from medical images, a large amount of quantitative data that, combined with pattern recognition procedures, allow for the resolution of many clinical issues with high accuracy. Examples of features used in the oncology field are tumor size and shape, as well as intensity, statistical and textural metrics [15,16,17,18,19,20,21,22,23,24,25,26,27,28,29,30,31,32,33,34,35,36,37,38,39,40,41,42].

In this study, we designed several classifiers with the aim of discriminating between benign and malignant breast lesions using, as predictors, radiomic metrics extracted from CEM and DCE-MRI images. In order to optimize the analysis, balancing and feature selection procedures were performed.

## 2. Methods

### 2.1. Patient Selection

Patients were enrolled in this study, which was approved by the local ethical committee of National Cancer Institute of Naples Pascale Foundation. Fifty-four patients (mean age 54.3, range 31–78 years) with 79 histo-pathologically proven breast lesions (48 malignant lesions and 31 benign lesions) (Table 1) underwent both CEM and DCE-MRI. The lesions were retrospectively analyzed with radiomic and artificial intelligence approaches. Breast lesions were categorized based on the American Joint Committee on Cancer staging.

All women gave their written informed consent according to local ethical committee regulations.

Inclusion criteria: patient with known, histologically proven breast lesions who underwent both dual-energy CEM in craniocaudal (CC) and mediolateral oblique (MLO) views and DCE-MRI.

Exclusion criteria were previously reported in [43,44].

### 2.2. Imaging Protocol

CEM was acquired with the dual-energy mammography system (Hologic’s Selenia^®^ Dimensions^®^ Unit, Bedford, MA, USA) as reported in our previous studies [43]. Two minutes after the administration of 1.5 mL/kg body weight of iodinated contrast medium (Visipaque 320; GE Healthcare, Inc., Princeton, NJ, USA) at a rate of 2–3 mL/s, each woman was placed in a CC view. Four and eight minutes after administration of the contrast agent, each breast was compressed in the MLO view: early MLO and late MLO views, respectively.

DCE-MRI was acquired with a 1.5T MR scanner (Magnetom Symphony; Siemens Medical System, Erlangen, Germany) equipped with a dedicated breast coil with 16 channels. Scan settings are reported in our previous study [44]: one series before and nine series after the automatic intravenous injection of 0.1 mmol/kg body weight of a positive paramagnetic contrast material (Gd-DOTA; Dotarem, Guerbet, Roissy CdG CEDEX, France) were acquired.

### 2.3. Image Processing

Regions of interest were manually segmented, slice by slice, by two expert radiologists, with 25 and 20 years of experience in breast imaging, respectively.

Breast lesions were segmented on dual-energy subtracted images, where contrast uptake was emphasized, both in CC and in MLO, and on the third T1-weighted subtracted series where contrast uptake was emphasized.

Radiomics features were extracted using the Texture Toolbox of MATLAB^®^, realized by Vallières et al. [45], which includes 48 parameters calculated according to the Image Biomarker Standardization Initiative [46], as previously described in [43,44]. The textural features include both first-order and second-order features; an extra detailed description of each feature has been provided in Appendix A.

### 2.4. Statistical Analysis

The statistical analysis was performed with RStudio software [47].

To assess variability among radiomic feature values, the intra-class correlation coefficient (ICC) was calculated. A non-parametric Wilcoxon–Mann–Whitney test and receiver operating characteristic (ROC) analysis were performed and the Youden index was calculated to obtain the optimal cut off value for each feature; then, in order to assess analysis results, the area under the ROC curve (AUC), sensitivity (SENS), specificity (SPEC), positive predictive value (PPV), negative predictive value (NPV) and accuracy (ACC) were computed.

Linear classifier (linear discriminant analysis—LDA), decision tree (TREE), k-nearest neighbors (KNN), artificial neural network (NNET) and support vector machine (SVM) using all extracted metrics of textural parameters were used [14]. Configuration settings for each classifier are provided in our previous study [41,43]. The 10-fold cross validation (10-fold CV) and the leave-one-out cross validation (LOOCV) approaches and median values of AUC, accuracy, sensitivity, specificity, PPV and NPV were obtained.

Feature selection with the least absolute shrinkage and selection operator (LASSO) method [48] was performed considering both the λ value with the minimum mean squared error (minMSE) and the largest λ value within one standard error of it (1SE) [49].

In addition, the self-adaptive synthetic over-sampling (SASYNO) approach and the adaptive synthetic sampling (ADASYN) approach, to help balance the classes (malignant and benign), were used [50,51,52,53,54,55].

The best model was chosen considering the highest area under the ROC curve and highest accuracy.

A *p*-value < 0.05 was considered as significant.

## 3. Results

The time interval between CEM and DCE-MRI was 2.5 days as a median value (range 1–16 days).

Table 2 reports the diagnostic performance of significant textural parameters for DCE-MRI and for dual-energy CEM in all views (i.e., CC, early and late MLO view), expressed in terms of AUC and *p*-value. The best result, considering the single feature in a univariate approach, was reached by the energy, range and GLN_GLRLM extracted on DCE-MRI volume with an AUC of 0.72.

**Figure 1:**
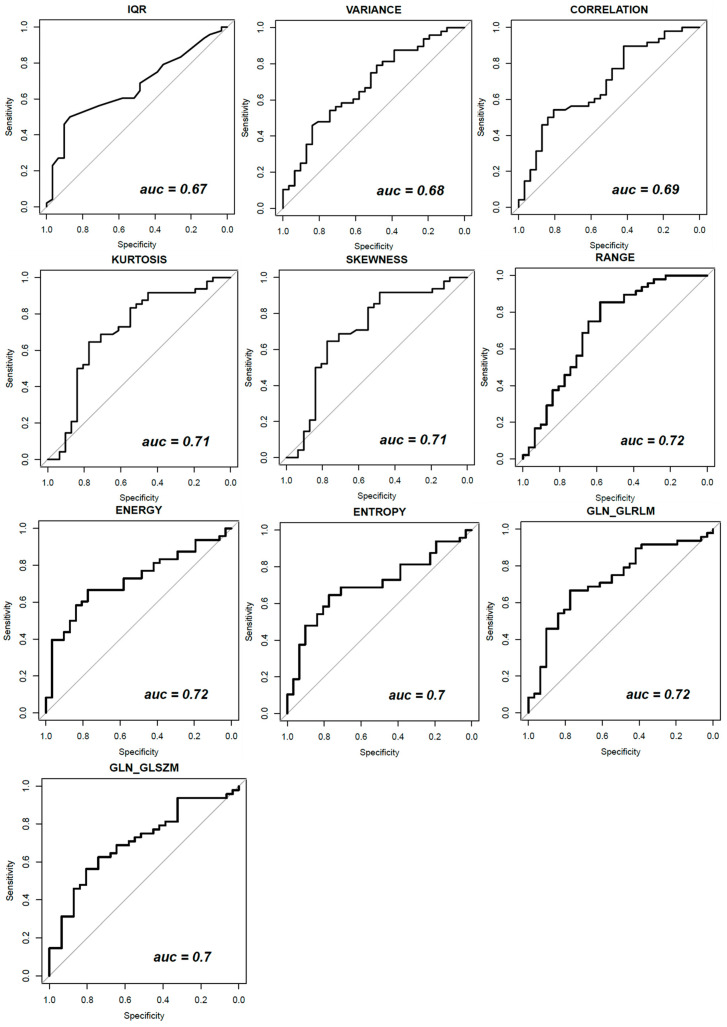
ROC curve trends of significant textural features for DCE-MRI and for dual-energy CEM CC, early and late MLO view.

Figure 1 shows ROC curve trends of significant textural features: variance, correlation and IQR for mammography CC projection, kurtosis and skewness for mammography early-MLO projection and range, energy, entropy, GLN_GLRLM and GLN_GLSZM for DCE-MRI images.

**Figure 2:**
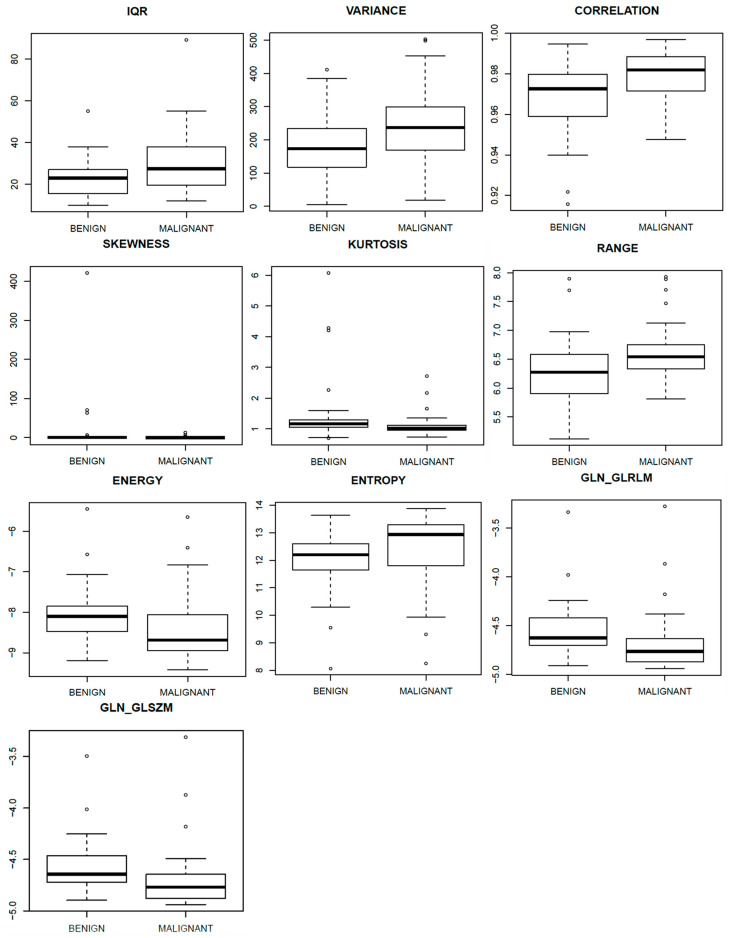
Boxplots of significant textural features for DCE-MRI and for dual-energy CEM CC, early and late MLO view.

Figure 2 shows the boxplots related to the above-mentioned parameters, to separate benign from malignant lesions.

Table 3 reports the performance achieved by the best classifiers designed to discriminate between benign and malignant lesions using CEM and DCE-MRI images.

The best performance, considering the CC view (ACC = 0.71; SENS = 0.71; SPEC = 0.71; PPV = 0.71; NPV = 0.71; AUC = 0.77), was reached with an SVM trained with 10-fold CV and balanced data (with SASYNO function) and a subset of four features (by LASSO and λminMSE). The subset of four robust textural features includes IQR, VARIANCE, CORRELATION and RLV.

The best performance, considering the early-MLO view (ACC = 0.76; SENS = 0.65; SPEC = 0.87; PPV = 0.82; NPV = 0.74; AUC = 0.73), was reached with an LDA trained with 10-fold CV and trained with balanced data (with ADASYN function) and all 48 textural features.

The best performance, considering the late-MLO view (ACC = 0.75; SENS = 0.71; SPEC = 0.77; PPV = 0.72; NPV = 0.75; AUC = 0.80), was reached with an LDA trained with 10-fold CV and balanced data (with ADASYN function) and a subset of 17 features (by LASSO). The subset of 17 robust textural features includes MEAN, MODE, MAD, RANGE, VARIANCE, CONTRAST, CORRELATION, SRLGE, LRLGE, RLV, SZE, SZLGE, SZHGE, GLV_GLSZM, BUSYNESS, COMPLEXITY and STRENGTH.

The best performance, considering all three mammographic projections (ACC = 0.79; SENS = 0.75; SPEC = 0.81; PPV = 0.78; NPV = 0.79; AUC = 0.81), was reached with an NNET trained with LOOCV and balanced data (with ADASYN function) and a subset of 15 features (by LASSO). The subset of 15 robust textural features includes IQR, VARIANCE, CORRELATION, LRHGE, GLV_GLRLM and SZLGE among textural features extracted from CC view; MODE, CONTRAST and GLV_GLRLM among textural features extracted from early-MLO view; MODE, STD, RANGE, IQR, CORRELATIOND and COMPLEXITY among textural features extracted from late-MLO view.

With regard to DCE-MRI images, the best performance (ACC = 0.74; SENS = 0.73; SPEC = 0.75; PPV = 0.74; NPV = 0.73; AUC = 0.72) was reached with an SVM trained with 10-fold CV and balanced data (with SASYNO function) and a subset of 15 features (by LASSO and λminMSE). The subset of 15 robust textural features includes MODE, MEDIAN, STD, MAD, ENTROPY, SUM AVERAGE, SRE, GLN_GLRLM, SRHGE, LZE, ZSN, ZP, LZHGE, GLV_GLSZM and ZSV.

Table 4 reports the performance achieved by the best classifiers to discriminate benign from malignant lesions when features extracted from CEM and DCE-MRI were combined. The best results overall (ACC = 0.84; SENS = 0.73; SPEC = 0.92; PPV = 0.90; NPV = 0.79; AUC = 0.88) were obtained considering a subset of 18 textural features extracted from all three mammographic views (CC, early MLO and late MLO) and DCE-MRI with an LDA trained with 10-fold CV and with balanced data (with ADASYN function). The subset of 18 robust textural features (by LASSO and λminMSE) includes IQR, VARIANCE, CORRELATION, LRHGE, GLV_GLRLM and RLV among textural features extracted from CC mammographic view; MODE and CONTRAST among textural features extracted from early-MLO mammographic view; STD, RANGE, CORRELATION and COMPLEXITY among textural features extracted from late-MLO mammographic view; RANGE, KURTOSIS, AUTOCORRELATION, LRHGE, LZE and GLV_GLSZM among textural features extracted from DCE-MRI images.

**Figure 3:**
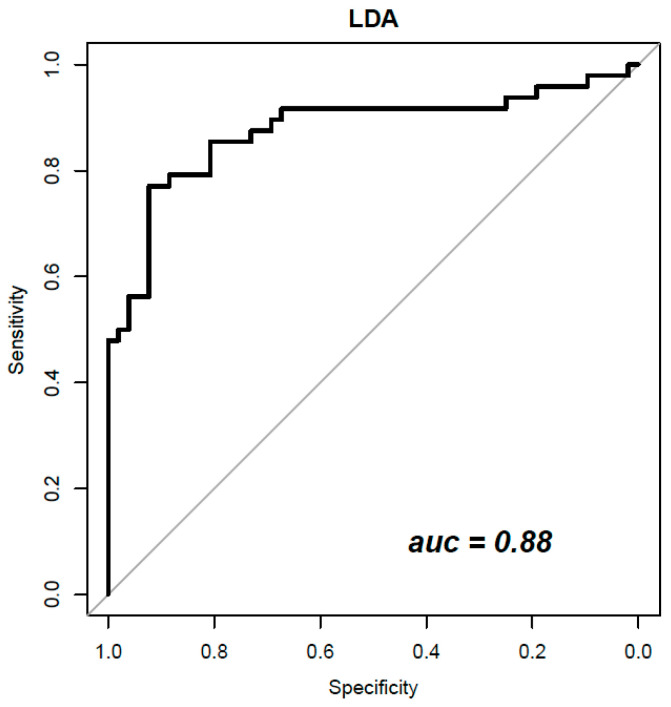
LDA classifier ROC curve trained with 18 robust radiomic features from CEM and DCE-MRI.

Figure 3 shows the ROC curves of the best classifier obtained combining features from CEM and DCE-MRI.

## 4. Discussion

Using texture features from dual-energy CEM and DCE-MRI, considered both individually and in combination, we aimed to evaluate radiomic analysis in discriminating between malignant and benign breast lesions.

In recent years, many studies have addressed the problem of breast lesion classification by using several feature categories such as textural and morphological features, in combination with different machine learning approaches, based on CEM and on DCE-MRI images analysis considered separately [30,31,32,33,34,35,36,37,38,39,40,41,56,57,58,59,60].

Marino et al. [61] investigated the potential of radiomic analysis of both CEM and DCE-MRI of the breast for the non-invasive assessment of tumor invasiveness, hormone receptor status and tumor grade in patients with primary breast cancer. This retrospective study included 48 female patients with 49 biopsy-proven breast cancers who underwent pretreatment breast CEM and MRI. Radiomic analysis was performed by using MaZda software. Radiomic parameters were correlated with tumor histology (invasive vs. non-invasive), hormonal status (HR+ vs. HR−) and grading (low grade G1 + G2 vs. high grade G3). CEM radiomics analysis yielded classification accuracies of up to 92% for invasive vs. non-invasive breast cancers, 95.6% for HR+ vs. HR− breast cancers and 77.8% for G1 + G2 vs. G3 invasive cancers. MRI radiomics analysis yielded classification accuracies of up to 90% for invasive vs. non-invasive breast cancers, 82.6% for HR+ vs. HR− breast cancers and 77.8% for G1 + G2 vs. G3 cancers. Their study, however, did not reported the combination of radiomic features extracted from CEM and DCE-MRI.

Jiang et al. [62] noninvasively evaluated the use of intratumoral and peritumoral regions from full-field digital mammography (DM), digital breast tomosynthesis (DBT) and dynamic contrast-enhanced and diffusion-weighted (DW) magnetic resonance imaging images separately and combined to predict the Ki-67 level based on radiomics. Their results demonstrated that the combined intra- and peritumoral radiomic signatures improved the AUC compared with the intra- or peritumoral radiomic signature in each modality. The nomogram incorporating the multi-model radiomics signature, age and lymph node metastasis status achieved the best prediction performance in the training (AUC = 0.922) and validation (AUC = 0.866) cohorts.

Zhao et al. [63] constructed radiomic models from DCE-MRI and mammography for the values in the diagnosis of breast cancer, reporting an accuracy of the individual model of 83.2% for DCE-MRI, 75.7% for mammography lesion, 64.4% for mammography margin and 77.2% for lesion + margin. When all features were combined, the accuracy increased to 89.6%.

Niu et al. [64] evaluated digital mammography, DBT, DCE- and DW-MRI, individually and combined, for the values in the diagnosis of breast cancer. They reported that the radiomic signature derived from DBT plus DM generated a lower AUC and sensitivity, but a higher specificity compared with that from DCE plus DWI. The nomogram integrating the combined radiomic signature, age and menstruation status achieved the best diagnostic performance in the training (AUC = 0.975) and validation (AUC = 0.983) cohorts.

Our results demonstrated that, considering the single metrics extracted from CEM, the best predictors were KURTOSIS (area under ROC curve (AUC) = 0.71) and SKEWNESS (AUC = 0.71) calculated on late MLO view. Considering the features calculated from DCE-MRI, the best predictors were RANGE (AUC = 0.72), ENERGY (AUC = 0.72), ENTROPY (AUC = 0.70) and GLN (Gray-Level Nonuniformity) of the gray-level run-length matrix (AUC = 0.72).

Considering the analysis with classifiers and the unbalanced dataset, no significant results were obtained. After the balancing and feature selection procedures, higher values of accuracy, specificity and AUC were reached. The best performance was obtained considering 18 robust features among all metrics derived from CEM and DCE-MRI, using a linear discriminant analysis (accuracy of 0.84 and AUC = 0.88).

This study had some limitations. The small cohort of studied patients represents a preliminary result to validate increasing the cohort of patients. Manual segmentation was time-consuming and could be operator-dependent and lose reproducibility; however, an automatic segmentation considering possible multicentric lesions or background parenchymal enhancement could be difficult to perform. In this study, the histological differences of tumors were not considered. This could improve the performance in the classification problem and allow for the classification of breast lesions according to grading and histotype.

Both DCE-MRI and CEM provide functional information on neoplastic neo-angiogenesis. CEM is an attractive alternative when MRI is not available, contraindicated or poorly tolerated. However, at our institution, a study protocol to compare DCE-MRI and CEM in staging and follow-up in breast cancer is still ongoing. Therefore, a future endpoint could be to design separate classifiers for CEM and DCE-MRI images and then merge the results in specific clinical settings, such as during patient follow-up in cases of suspicious local recurrence.

## 5. Conclusions

In conclusion, classifiers adjusted with adaptive synthetic sampling and feature selection allowed for increased diagnostic performance of CEM and DCE-MRI in the differentiation between benign and malignant lesions.

## Figures and Tables

**Table 1:** Number and corresponding percentage of the total benign or malignant breast lesions.

**Benign (31 Lesions)**	**Number**	**Percentage Value (%)**
Fibrosis	6	19.35
Ductal hyperplasia	8	25.81
Fibroadenoma	9	29.03
Dysplasia	4	12.90
Adenosis	4	12.90
**Malignant (48 Lesions)**	**Number**	**Percentage Value (%)**
Infiltrating lobular carcinoma	7	14.58
Infiltrating ductal carcinoma	30	62.50
Ductal carcinoma in situ	9	18.75
Tubular Carcinoma	2	4.17

**Table 2:** Accuracy of significant textural parameters for DCE-MRI and for dual-energy CEM CC, early and late MLO view.

Mammography Projection	Textural Parameters	AUC Values	*p*-Value
CC-view	IQR	0.67	0.01
	Variance	0.68	0.01
	Correlation	0.69	0.000
MLO view	Kurtosis	0.71	0.000
	Skewness	0.71	0.000
Magnetic Resonance Images	Textural Parameters	AUC Values	*p*-Value
	Range	0.72	0.001
	Energy	0.72	0.001
	Entropy	0.70	0.003
	GLN_GLRLM	0.72	0.001
	GLN_GLSZM	0.70	0.002

**Table 3:** Performance of the best classifiers designed to discriminate between benign and malignant lesions.

Classifier	Cross-Validation	ACC	SENS	SPEC	PPV	NPV	AUC
***CEM–CC*** **view**							
Performance of classifiers trained with balanced data (with ADASYN function) and a subset of 34 textural features (AUC ≥ 0.60).							
TREE	10-fold CV	0.74	0.74	0.78	0.76	0.74	0.73
Performance of classifiers trained with balanced data (with SASYNO function) and a subset of 4 robust textural features (by LASSO and λ_minMSE_).							
LDA	10-fold CV	0.71	0.71	0.71	0.71	0.71	0.76
	LOOCV	0.71	0.71	0.71	0.71	0.71	0.75
SVM	10-fold CV	0.71	0.71	0.71	0.71	0.71	0.77
Performance of classifiers trained with balanced data (with SASYNO function) and a subset of 3 robust textural features (by LASSO and λ_1SE_).							
LDA	10-fold CV	0.71	0.71	0.71	0.71	0.71	0.76
	LOOCV	0.71	0.71	0.71	0.71	0.71	0.75
NNET	10-fold CV	0.70	0.71	0.69	0.69	0.70	0.74
	LOOCV	0.70	0.73	0.67	0.69	0.71	0.74
SVM	10-fold CV	0.71	0.71	0.71	0.71	0.71	0.75
	LOOCV	0.72	0.73	0.71	0.71	0.72	0.76
***CEM–early MLO view***							
Performance of classifiers trained with balanced data (with ADASYN function) and all 48 textural features							
LDA	10-fold CV	0.76	0.65	0.87	0.82	0.74	0.73
	LOOCV	0.75	0.60	0.87	0.81	0.72	0.71
Performance of classifiers trained with balanced data (with ADASYN function) and a subset of 7 robust textural features (by LASSO and λ_minMSE_).							
LDA	10-fold CV	0.66	0.54	0.75	0.65	0.65	0.72
	LOOCV	0.66	0.56	0.75	0.66	0.66	0.7
Performance of classifiers trained with balanced data (with ADASYN function) and a subset of 14 robust textural features (by LASSO and λ_1SE_).							
LDA	10-fold CV	0.62	0.52	0.69	0.60	0.62	0.71
	LOOCV	0.66	0.56	0.75	0.66	0.66	0.7
***CEM–late MLO view***							
Performance of classifiers trained with balanced data (with ADASYN function) and all 48 textural features							
LDA	10-fold CV	0.78	0.71	0.84	0.79	0.77	0.78
	LOOCV	0.78	0.69	0.86	0.80	0.76	0.77
Performance of classifiers trained with balanced data (with ADASYN function) and a subset of 17 robust textural features (by LASSO and λ_minMSE_).							
LDA	10-fold CV	0.75	0.71	0.77	0.72	0.75	0.8
	LOOCV	0.73	0.71	0.75	0.71	0.75	0.8
NNET	10-fold CV	0.72	0.65	0.77	0.70	0.72	0.78
	LOOCV	0.72	0.69	0.75	0.70	0.74	0.72
Performance of classifiers trained with balanced data (with ADASYN function) and a subset of 14 robust textural features (by LASSO and λ_1SE_).							
LDA	10-fold CV	0.71	0.69	0.71	0.67	0.73	0.78
	LOOCV	0.70	0.69	0.71	0.67	0.73	0.78
NNET	10-fold CV	0.71	0.67	0.75	0.70	0.72	0.74
	LOOCV	0.74	0.69	0.79	0.73	0.75	0.74
***CEM–CC + early MLO + late view***							
Performance of classifiers trained with balanced data (with ADASYN function) and a subset of 15 robust textural features (by LASSO and λ_minMSE_).							
LDA	10-fold CV	0.75	0.69	0.81	0.77	0.75	0.82
	LOOCV	0.76	0.71	0.81	0.77	0.76	0.81
NNET	10-fold CV	0.77	0.75	0.80	0.77	0.78	0.79
	LOOCV	0.79	0.75	0.81	0.78	0.79	0.81
SVM	10-fold CV	0.72	0.73	0.70	0.69	0.75	0.79
	LOOCV	0.76	0.73	0.80	0.76	0.77	0.81
Performance of classifiers trained with balanced data (with ADASYN function) and a subset of 8 robust textural features (by LASSO and λ_1SE_).							
NNET	10-fold CV	0.72	0.73	0.72	0.70	0.75	0.78
***DCE-MRI***							
Performance of classifiers trained with balanced data (with ADASYN function) and all 48 textural features							
LDA	10-fold CV	0.73	0.69	0.77	0.73	0.73	0.71
	LOOCV	0.70	0.65	0.75	0.70	0.70	0.7
Performance of classifiers trained with balanced data (with SASYNO function) and a subset of 15 robust textural features (by LASSO and λ_minMSE_).							
SVM	10-fold CV	0.74	0.73	0.75	0.74	0.73	0.72
	LOOCV	0.70	0.69	0.71	0.70	0.69	0.71

**Table 4:** Performance achieved by the best classifiers to discriminate between benign and malignant lesions for combined CEM and DCE-MRI.

Classifier	Cross-Validation	ACC	SENS	SPEC	PPV	NPV	AUC
Performance for classifiers trained with balanced data (with ADASYN function) and a subset of 18 robust textural features (by LASSO and λ_minMSE_).							
LDA	10-fold CV	0.84	0.73	0.92	0.90	0.79	0.88
	LOOCV	0.80	0.71	0.88	0.85	0.77	0.87
SVM	10-fold CV	0.84	0.81	0.87	0.85	0.83	0.86
	LOOCV	0.83	0.79	0.87	0.84	0.82	0.86
Performance for classifiers trained with balanced data (with SASYNO function) and a subset of 3 robust textural features (by LASSO and λ_minMSE_).							
LDA	10-fold CV	0.79	0.79	0.79	0.79	0.79	0.88
	LOOCV	0.79	0.79	0.79	0.79	0.79	0.89
SVM	10-fold CV	0.80	0.79	0.79	0.79	0.79	0.86
	LOOCV	0.79	0.77	0.81	0.80	0.78	0.87

## Data Availability

Data are available at link https://zenodo.org/record/6344730#.YixvazXSK3A (accessed on 20 January 2022).

## Appendix A. Definition of Textural Features

### Appendix A.1. First-Order Gray-Level Statistics

First-order gray-level statistics describe the distribution of gray values within the volume. Let *X* denote the 3-D image matrix with *N* voxels, *P* the first order histogram, *P*(*i*) the fraction of voxels with intensity level *i* and *N*l the number of discrete intensity levels.

- Mean, the mean gray level of X.
  $$mean=\frac{1}{N}\overset{N}{\underset{i=1}{\sum }}X\left(i\right)$$

- Mode, the most frequent element(s) of array X.

- Median, the sample median of X or the 50th percentile of X.

- Standard deviation (STD)
  $$STD={\left(\frac{1}{N-1}\overset{N}{\underset{i=1}{\sum }}{\left(X\left(i\right)-\bar{X}\right)}^{2}\right)}^{1/2}$$

- Mean Absolute Deviation (MAD), the mean of the absolute deviation of all voxel intensities around the mean intensity value.
  $$MAD=\frac{1}{N}\overset{N}{\underset{i=1}{\sum }}\left|X\left(i\right)-\bar{X}\right|$$

- Range, the range of intensity values of X.
  range=max(X)−min(X)
  where max(X) is the maximum intensity value of X and min(X) is the minimum intensity value of X.

- Interquartile range (IQR), the interquartile range is defined as the 75th minus the 25th percentile of X.

- Kurtosis:
  $$kurtosis=\frac{\frac{1}{N}\sum_{i=1}^{N}{\left(X\left(i\right)-\bar{X}\right)}^{4}}{{\left(\sqrt{\frac{1}{N}\sum_{i=1}^{N}{\left(X\left(i\right)-\bar{X}\right)}^{2}}\right)}^{2}}$$
  where $\bar{X}$ is the mean of X.

- Variance, Variance is the square of the standard deviation:
  $$variance=\frac{1}{N-1}\overset{N}{\underset{i=1}{\sum }}{\left(X\left(i\right)-\bar{X}\right)}^{2}$$
  where $\bar{X}$ is the mean of X.

- Skewness:
  $$skewness=\frac{\frac{1}{N}\sum_{i=1}^{N}{\left(X\left(i\right)-\bar{X}\right)}^{3}}{{\left(\sqrt{\frac{1}{N}\sum_{i=1}^{N}{\left(X\left(i\right)-\bar{X}\right)}^{2}}\right)}^{3}}$$
  where $\bar{X}$ is the mean of X.

### Appendix A.2. Gray Level Co-Occurrence Matrix (GLCM)

A normalized GLCM is defined as P(i,j;δ,α), a metric with size $N_{g}\times N_{g}$ describing the second-order joint probability function of an image, where the (i,j)th element represents the number of times the combination of intensity levels i and j occur in two pixels in the image, that are separated by a distance of δ pixels in direction α and $N_{g}$ is the maximum discrete intensity level in the image. Let:

- P(i,j) be the normalized (i.e., $\sum^{\;}P\left(i,j\right)=1$) co-occurrence matrix, generalized for any δ and α,
- $p_{x}\left(i\right)\;=\;\sum_{j=1}^{N_{g}}P\left(i,j\right)$,
- $p_{y}\left(j\right)\;=\;\sum_{i=1}^{N_{g}}P\left(i,j\right)$,
- $\mu_{x}$ be the mean of $p_{x}$, where $\mu_{x}=\;\sum_{i=1}^{N_{g}}\sum_{j=1}^{N_{g}}iP\left(i,j\right)$,
- $\mu_{y}$ be the mean of $p_{y}$, where $\mu_{y}=\;\sum_{i=1}^{N_{g}}\sum_{j=1}^{N_{g}}jP\left(i,j\right)$,
- $\sigma_{x}$ be the standard deviation of $p_{x}$, where $\sigma_{x}=\;\sum_{i=1}^{N_{g}}\sum_{j=1}^{N_{g}}P\left(i,j\right){\left(i-\mu_{x}\right)}^{2}$,
- $\sigma_{y}$ be the standard deviation of $p_{y}$, where $\sigma_{y}=\;\sum_{i=1}^{N_{g}}\sum_{j=1}^{N_{g}}P\left(i,j\right){\left(j-\mu_{y}\right)}^{2}$.

- Energy
  $$energy=\overset{N_{g}}{\underset{i=1}{\sum }}\overset{N_{g}}{\underset{j=1}{\sum }}{\left[P\left(i,j\right)\right]}^{2}$$

- Contrast
  $$contrast=\overset{N_{g}}{\underset{i=1}{\sum }}\overset{N_{g}}{\underset{j=1}{\sum }}{\left|i-j\right|}^{2}P\left(i,j\right)$$

- Entropy
  $$entropy=-\overset{N_{g}}{\underset{i=1}{\sum }}\overset{N_{g}}{\underset{j=1}{\sum }}P\left(i,j\right)log_{2}\left[P\left(i,j\right)\right]$$

- Homogeneity
  $$homogeneity=\overset{N_{g}}{\underset{i=1}{\sum }}\overset{N_{g}}{\underset{j=1}{\sum }}\frac{P\left(i,j\right)}{1+\left|i-j\right|}\;$$

- Correlation
  $$correlation=\frac{\sum_{i=1}^{N_{g}}\sum_{j=1}^{N_{g}}ijP\left(i,j\right)-\mu_{x}\mu_{y}}{\sigma_{x}\sigma_{y}}$$

- Sum Average
  $$sum\;average=\frac{1}{N_{g}\times N_{g}}\overset{N_{g}}{\underset{i=1}{\sum }}\overset{N_{g}}{\underset{j=1}{\sum }}\left[iP\left(i,j\right)+jP\left(i,j\right)\right]$$

- Dissimilarity
  $$dissimilarity=\overset{N_{g}}{\underset{i=1}{\sum }}\overset{N_{g}}{\underset{j=1}{\sum }}\left|i-j\right|P\left(i,j\right)\;$$

- Autocorrelation
  $$autocorrelation=\overset{N_{g}}{\underset{i=1}{\sum }}\overset{N_{g}}{\underset{j=1}{\sum }}ijP\left(i,j\right)\;$$

### Appendix A.3. Gray Level Run-Length Matrix (GLRLM)

Run-length metrics quantify gray level runs in an image. A gray level run is defined as the length in number of pixels, of consecutive pixels that have the same gray level value. In a gray level run length matrix p(i,j|θ), the (i,j)th element describes the number of times *j* a gray level *i* appears consecutively in the direction specified by *θ*. Let:

- p(i,j) be the (i,j)th entry in the given run-length matrix *p*, generalized for any direction *θ*,
- $N_{g}$ be the number of discrete intensity values in the image,
- $N_{r}$ be the maximum run length,
- $N_{s}$ be the total numbers of runs, where $N_{s}=\;\overset{N_{g}}{\underset{i=1}{\sum }}\overset{N_{r}}{\underset{j=1}{\sum }}p\left(i,j\right)$,
- $p_{r}$ be the sum distribution of the number of runs with run length j, where $p_{r}\left(j\right)=\overset{N_{g}}{\underset{i=1}{\sum }}p\left(i,j\right)$,
- $p_{g}$ be the sum distribution of the number of runs with run length i, where $p_{g}\left(i\right)=\overset{N_{r}}{\underset{j=1}{\sum }}p\left(i,j\right)$,
- $N_{p}$ be the number of voxels in the image, where $N_{p}=\overset{N_{r}}{\underset{j=1}{\sum }}jp_{r}$,
- $\mu_{r}$ be the mean run length, where $\mu_{r}=\;\overset{N_{g}}{\underset{i=1}{\sum }}\overset{N_{r}}{\underset{j=1}{\sum }}jp_{n}\left(i,j\right)$,
- $\mu_{g}$ be the mean gray level, where $\mu_{g}=\;\overset{N_{g}}{\underset{i=1}{\sum }}\overset{N_{r}}{\underset{j=1}{\sum }}ip_{n}\left(i,j\right)$.

- Short-Run Emphasis (SRE)
  $$SRE=\overset{N_{r}}{\underset{j=1}{\sum }}\frac{p_{r}}{j^{2}}$$

- Long-Run Emphasis (LRE)
  $$LRE=\overset{N_{r}}{\underset{j=1}{\sum }}j^{2}p_{r}$$

- Gray Level Nonuniformity (GLN)
  $$GLN=\overset{N_{g}}{\underset{i=1}{\sum }}p_{g}{}^{2}$$

- Run-Length Nonuniformity (RLN)
  $$RLN=\overset{N_{r}}{\underset{j=1}{\sum }}p_{r}{}^{2}$$

- Run Percentage (RP)
  $$RP=\frac{N_{s}}{N_{p}}$$

- Low Gray Level Run Emphasis (LGRE)
  $$LGRE=\overset{N_{g}}{\underset{i=1}{\sum }}\frac{p_{g}}{i^{2}}$$

- High Gray Level Run Emphasis (HGRE)
  $$HGRE=\overset{N_{g}}{\underset{i=1}{\sum }}i^{2}p_{g}$$

- Short-Run Low Gray Level Emphasis (SRLGE)
  $$SRLGE=\overset{N_{g}}{\underset{i=1}{\sum }}\overset{N_{r}}{\underset{j=1}{\sum }}\frac{p\left(i,j\right)}{i^{2}j^{2}}$$

- Short-Run High Gray Level Emphasis (SRHGE)
  $$SRHGE=\overset{N_{g}}{\underset{i=1}{\sum }}\overset{N_{r}}{\underset{j=1}{\sum }}\frac{p\left(i,j\right)i^{2}}{j^{2}}$$

- Long-Run Low Gray Level Emphasis (LRLGE)
  $$LRLGE=\overset{N_{g}}{\underset{i=1}{\sum }}\overset{N_{r}}{\underset{j=1}{\sum }}\frac{p\left(i,j\right)j^{2}}{i^{2}}$$

- Long-Run High Gray Level Emphasis (LRHGE)
  $$LRHGE=\overset{N_{g}}{\underset{i=1}{\sum }}\overset{N_{r}}{\underset{j=1}{\sum }}p\left(i,j\right)i^{2}j^{2}$$

- Gray Level Variance (GLV)
  $$GLV=\frac{1}{N_{g}\times N_{r}}\;\overset{N_{g}}{\underset{i=1}{\sum }}\overset{N_{r}}{\underset{j=1}{\sum }}{\left(ip\left(i,j\right)-\mu_{g}\right)}^{2}$$

- Run-Length Variance (RLV)
  $$RLV=\frac{1}{N_{g}\times N_{r}}\;\overset{N_{g}}{\underset{i=1}{\sum }}\overset{N_{r}}{\underset{j=1}{\sum }}{\left(jp\left(i,j\right)-\mu_{r}\right)}^{2}$$

### Appendix A.4. Gray Level Size Zone Matrix (GLSZM)

A gray level size-zone matrix describes the amount of homogeneous connected areas within the volume, of a certain size and intensity. The (i,j) entry of the GLSZM p(i,j) is the number of connected areas of gray level (i.e., intensity value) *i* and size *j*. GLSZM features therefore describe homogeneous areas within the tumor volume, describing tumor heterogeneity at a regional scale [5]. Let:

- p(i,j) be the (i,j)th entry in the given GLSZM  p,
- $N_{g}$ be the number of discrete intensity values in the image,
- $N_{z}$ be the size of the largest, homogeneous region in the volume of interest,
- $N_{s}$ be the total number of homogeneous regions (zones), where $N_{s}=\;\overset{N_{g}}{\underset{i=1}{\sum }}\overset{N_{z}}{\underset{j=1}{\sum }}p\left(i,j\right)$,
- $p_{z}$ be the sum distribution of the number of zones with size j, where $p_{z}\left(j\right)=\overset{N_{g}}{\underset{i=1}{\sum }}p\left(i,j\right)$,
- $p_{g}$ be the sum distribution of the number of zones with gray level i, where $p_{g}\left(i\right)=\overset{N_{z}}{\underset{j=1}{\sum }}p\left(i,j\right)$,
- $N_{p}$ be the number of voxels in the image, where $N_{p}=\overset{N_{z}}{\underset{j=1}{\sum }}jp_{r}$,
- $\mu_{r}$ be the mean zone size, where $\mu_{r}=\;\overset{N_{g}}{\underset{i=1}{\sum }}\overset{N_{z}}{\underset{j=1}{\sum }}jp(i,j|\theta )$,
- $\mu_{g}$ be the mean gray level, where $\mu_{g}=\;\overset{N_{g}}{\underset{i=1}{\sum }}\overset{N_{z}}{\underset{j=1}{\sum }}ip(i,j|\theta )$.

- Small Zone Emphasis (SZE)
  $$SZE=\overset{N_{z}}{\underset{j=1}{\sum }}\frac{p_{z}}{j^{2}}$$

- Large Zone Emphasis (LZE)
  $$LZE=\overset{N_{z}}{\underset{j=1}{\sum }}j^{2}p_{z}$$

- Gray Level Nonuniformity (GLN)
  $$GLN=\overset{N_{g}}{\underset{i=1}{\sum }}p_{g}{}^{2}$$

- Zone Size Nonuniformity (ZSN)
  $$ZSN=\overset{N_{g}}{\underset{i=1}{\sum }}p_{z}{}^{2}$$

- Zone Percentage (ZP)
  $$ZP=\frac{N_{s}}{N_{p}}$$

- Low Gray Level Zone Emphasis (LGZE)
  $$LGZE=\overset{N_{g}}{\underset{i=1}{\sum }}\frac{p_{g}}{i^{2}}$$

- High Gray Level Zone Emphasis (HGZE)
  $$HGZE=\overset{N_{g}}{\underset{i=1}{\sum }}i^{2}p_{g}$$

- Small Zone Low Gray Level Emphasis (SZLGE)
  $$SZLGE=\overset{N_{g}}{\underset{i=1}{\sum }}\overset{N_{z}}{\underset{j=1}{\sum }}\frac{p\left(i,j\right)}{i^{2}j^{2}}$$

- Small Zone High Gray Level Emphasis (SZHGE)
  $$SZHGE=\overset{N_{g}}{\underset{i=1}{\sum }}\overset{N_{z}}{\underset{j=1}{\sum }}\frac{p\left(i,j\right)i^{2}}{j^{2}}$$

- Large Zone Low Gray Level Emphasis (LZLGE)
  $$LZLGE=\overset{N_{g}}{\underset{i=1}{\sum }}\overset{N_{z}}{\underset{j=1}{\sum }}\frac{p\left(i,j\right)j^{2}}{i^{2}}$$

- Large Zone High Gray Level Emphasis (LZHGE)
  $$LZHGE=\overset{N_{g}}{\underset{i=1}{\sum }}\overset{N_{z}}{\underset{j=1}{\sum }}p\left(i,j\right)j^{2}i^{2}$$

- Gray Level Variance (GLV)
  $$GLV=\frac{1}{N_{g}\times N_{z}}\;\overset{N_{g}}{\underset{i=1}{\sum }}\overset{N_{z}}{\underset{j=1}{\sum }}{\left(ip\left(i,j\right)-\mu_{g}\right)}^{2}$$

- Zone Size Variance (ZSV)
  $$ZSV=\frac{1}{N_{g}\times N_{z}}\;\overset{N_{g}}{\underset{i=1}{\sum }}\overset{N_{z}}{\underset{j=1}{\sum }}{\left(jp\left(i,j\right)-\mu_{z}\right)}^{2}$$

### Appendix A.5. Neighborhood Gray Tone Difference Matrix (NGTDM)

The *i*th entry of the NGTDM s(i|d) is the sum of gray level differences of voxels with intensity *i* and the average intensity $A_{i}$ of their neighboring voxels within a distance *d*. Let:

- $n_{i}$ be the number of voxels with gray level i,
- $N=\sum^{\;}n_{i}$ be the total number of voxels,
- $s\left(i\right)=\;\{\begin{array}{c} \underset{n_{i}}{\sum }\left|i-\;A_{i}\right|\;for\;n_{i}>0\\ 0\;otherwise \end{array}$ be generalized for any distance d,
- $N_{g}$ be the maximum discrete intensity level in the image,
- $p\left(i\right)=\;\frac{n_{i}}{N}$ be the probability of gray level i,
- $N_{p}$ be the total number of gray levels present in the image.

- Coarseness:
  $$coarseness={\left[\varepsilon +\overset{N_{g}}{\underset{n=1}{\sum }}p\left(i\right)s\left(i\right)\right]}^{-1}$$
  where ε is a small number to prevent coarseness from becoming infinite.

- Contrast
  $$contrast=\left(\frac{1}{N_{p}\left(1-N_{p}\right)}\overset{N_{g}}{\underset{i=1}{\sum }}\overset{N_{g}}{\underset{j=1}{\sum }}p\left(i\right)p\left(j\right){\left(i-j\right)}^{2}\right)\left(\frac{1}{N}\overset{N_{g}}{\underset{i=1}{\sum }}s\left(i\right)\right)$$
- Busyness
  $$busyness=\frac{\sum_{i=1}^{N_{g}}p\left(i\right)s\left(i\right)}{\sum_{i=1}^{N_{g}}\sum_{j=1}^{N_{g}}\left|ip\left(i\right)-jp\left(j\right)\right|},\;\;\;\;\;\;\;\;\;\;\;\;\;\;\;\;\;\;\;\;\;\;\;\;\;\;\;\;\;\;\;\;\;\;p\left(i\right)\neq 0,\;p\left(j\right)\neq 0\;$$
- Complexity
  $$complexity=\overset{N_{g}}{\underset{i=1}{\sum }}\overset{N_{g}}{\underset{j=1}{\sum }}\left|i-j\right|\frac{p\left(i\right)s\left(i\right)+p\left(j\right)s\left(j\right)}{N\left(p\left(i\right)+p\left(j\right)\right)},\;\;\;\;\;\;\;\;\;\;\;\;\;\;p\left(i\right)\neq 0,\;p\left(j\right)\neq 0$$
- Strength
  $$strength=\frac{\sum_{i=1}^{N_{g}}\sum_{j=1}^{N_{g}}\left[p\left(i\right)+p\left(j\right)\right]{\left(i-j\right)}^{2}}{\varepsilon +\sum_{n=1}^{N_{g}}s\left(i\right)},\;\;\;\;\;\;\;\;\;\;\;\;\;\;\;\;\;\;\;\;\;\;\;\;\;\;p\left(i\right)\neq 0,\;p\left(j\right)\neq 0\;$$
  where ε is a small number to prevent strength from becoming infinite.
